# The Peripherin Gene Regulates the Migration of Bone Marrow Mesenchymal Stem Cells in Wuzhishan Mini Pigs

**DOI:** 10.1155/2020/8856388

**Published:** 2020-10-12

**Authors:** Zheng Feng, Wentong Li, Ying Xia, Hui Yu, Hua Li, Kui Li, Yulian Mu

**Affiliations:** ^1^Guangdong Provincial Key Laboratory of Animal Molecular Design and Precise Breeding, School of Life Science and Engineering, Foshan University, Foshan, 528231 Guangdong, China; ^2^Institute of Animal Sciences, Chinese Academy of Agricultural Sciences, Beijing 100193, China

## Abstract

Increasing the migratory capacity of the implanted mesenchymal stem cells (MSCs) is a major challenge in developing successful cell transplantation therapies. Nevertheless, the regulatory factors involved in the migration of BMMSCs remain largely unknown. In this study, we studied the role of the peripherin (PRPH) gene in regulating the ability of Wuzhishan mini pig (WZSP) BMMSCs to migrate in vitro. Four different shRNA vectors directed against PRPH were designed and transfected into BMMSCs. The vector with the best interference effect was chosen to be used in the following experiments. The expression level of PRPH in BMMSCs was determined by quantitative real-time PCR and western blot analysis. The migration capacity of the BMMSCs was estimated using a scratch assay, a transwell in vitro migration model assay, and filamentous actin staining. The results showed that shRNA-mediated knockdown of the expression of the PRPH gene in BMMSCs reduced the ability of these cells to migrate. Overall, these results illustrate that the PRPH gene regulates the migration of BMMSCs in the WZSP.

## 1. Introduction

Mesenchymal stem cells (MSCs), a unique population of multipotent adult progenitor cells that are derived from marrow tissues, umbilical cord blood, adipose tissue, dental tissues, adult muscle, and corneal stroma, were first isolated from bone marrow (BM) and subsequently found to differentiate and propagate in vitro [1–3]. As seed cells with a superior capacity for self-renewal, multilineage differentiation, and immunomodulation, MSCs have been used in early-stage clinical trials for their ability to modulate the immune system, heal tissue, and regenerate various tissue types and organs [4]. BMMSCs are a special kind of cell population with totipotency and the potential to differentiate into multiple cell types. Under appropriate conditions, BMMSCs can proliferate and differentiate into mesenchymal cells and continue to differentiate into various connective tissue cells, such as bone, cartilage, fat, fibroblast, and vascular endothelial cells; they can also differentiate towards endodermal or ectodermal derivatives [5, 6]. BMMSCs have multiple advantages that make them ideal for tissue engineering and transplantation: they are easily obtained, carry a reduced risk of immune rejection, have the potential to differentiate into multiple cell types, and can be used in multiple transplantation methods.

Porcine MSCs are increasingly being studied as a substitute for human MSCs because they are easy to manipulate and share similar properties with human MSCs. The Wuzhishan mini pig (WZSP), which is characterized by its small adult size, is a unique and highly inbred line bred in China. Its physiological and general biochemical indexes are close to those of human beings, and the heart and guts of WZSP are also similar to those of humans, which makes it an ideal animal model for studying human diseases and organ transplantation [7]. In addition, WZSP is considered useful for medical and veterinary research because of its small size [8].

Currently, cell migration, which is involved in many physiological phenomena including cell foraging, wound healing, and cancer metastasis, is an important topic in cytobiology. The adhesive and migratory properties of MSCs may influence their fate and efficacy when injected into the circulatory system as a therapy and also their behavior in situ when acting as endogenous repair or immunomodulatory cells [9]. Therefore, it is very important to understand the mechanism of MSC migration.

There is evidence that peripherin, which is a type III intermediate filament cytoskeletal protein encoded by the PRPH gene, may play a role in cell migration as well as in axonal transport [10, 11] and in repair after axonal damage [12, 13]. Wray et al. found that luteinizing hormone-releasing hormone (LHRH) cells migrate from the olfactory pit into the diencephalon via the peripherin-positive/N-CAM-negative fiber track [14]. In addition, peripherin is homologous to other cytoskeletal proteins such as vimentin that are related to migration [15]. From the above findings, we propose that PRPH regulates cell migration.

This study was aimed at investigating the effects of knockdown of PRPH on the migration ability of WZSP BMMSCs. A specific short hairpin RNA (shRNA) eukaryotic expression vector targeting PRPH was transfected into BMMSCs, and the effect on the PRPH expression was investigated by quantitative real-time PCR (qRT-PCR) and western blot analysis. In addition, the effect of knockdown of PRPH on the migration ability of BMMSCs from WZSP was investigated using a scratch assay, a transwell in vitro migration model assay, and filamentous actin (F-actin) staining. Our findings indicate that PRPH can regulate MSC migration, which contributes to our understanding of the mechanism underlying MSC migration.

## 2. Materials and Methods

### 2.1. Culture of Porcine BMMSCs

BMMSCs were isolated from the femur and tibia of a 42-day-old WZSP, and evaluation of cell characteristics by flow cytometry and analysis of adipogenic and osteogenic differentiation was performed as described previously [16]. The BMMSCs were cultured in the DMEM/F12 media (Gibco) supplemented with 10% fetal bovine serum (FBS, Gibco) and penicillin (50 U/mL)/streptomycin (50 *μ*g/mL) (FBS, Gibco). The cultures were incubated at 37°C in a 5% CO_2_ atmosphere. The medium was changed every other day. The cells were grown to 80% confluence and then trypsinized (0.1% trypsin, Gibco) at 37°C for 1 min, harvested, and subcultured (1 : 2 split) in new vessels.

### 2.2. Design and Assessment of Specific Short Hairpin RNAs Targeting the PRPH Gene

shRNAs directed against PRPH were used to knock down the pig peripherin expression in BMMSCs. Four different shRNA sequences directed against PRPH were designed based on the PRPH gene sequence from the NCBI Gene database (Gene ID 100152434) following the design principles for shRNA [17–19] and synthesized by Shanghai GenePharma Co., Ltd. (Table 1). The following expression vectors encoding different shRNAs were constructed: shRNA-PRPH-633, shRNA-PRPH-653, shRNA-PRPH-1242, and shRNA-PRPH-1302. In addition, a negative control (shRNA-NC) was also designed and synthesized; this control had no homology to the PRPH gene sequences and had the same composition of nucleic acids as the specific shRNA sequences. The vectors were transfected into BMMSCs from the fourth to fifth generation by electroporation using the X-treme GENE HP DNA transfection reagent following the manufacturer's protocol (Liposomal Transfection Reagent Kit, Roche). qRT-PCR was employed to detect the expression of PRPH mRNA 24 h after transfection. We verified the knockdown activity of the four shRNA constructs and selected the vector with the best interference effect (shRNA-PRPH-653) for use in subsequent experiments. This vector was digested with the restriction endonuclease ApaLI (New England Biolabs). The linearized shRNA vector with no specific transfection to the BMMSCs was named shRNA-NC. The BMMSC experimental group was treated with the shRNA-PRPH-653 transfection vector, and the control group was treated with shRNA-NC. G418 (200 *μ*g/mL) was added to the culture medium 24 h after transfection; then, the culture media were changed once every two days. After elimination of nontransfected cells and selection for 14 days, stable cell lines transfected with shRNA-NC or shRNA-PRPH-653 were obtained. All BMMSCs were observed under a fluorescence microscope.

### 2.3. RNA Extraction and qRT-PCR Assay

The total RNA from transfected cells was isolated using Trizol (Invitrogen) and the High Purity Total RNA Quick Extraction Kit (BioTeke) according to the manufacturer's protocols. Total RNA (1.5 *μ*g) from each sample was reverse transcribed into cDNA using the RevertAid First Strand cDNA Synthesis Kit (Thermo Scientific). qRT-PCR was performed using SYBR Select Master Mix (Life Technologies). The primers used for amplification of PRPH (forward: 5′-CATCGAGATCGCCACCTACC-3′, reverse: 5′-CCCATCCCGGGTCTCAATAG-3′) and GAPDH (forward: 5′-GTGAAGGTCGGAGTGAACG-3′, reverse: 5′-CTCGCTCCTGGAAGATGGTG-3′) were designed and synthesized by Shanghai Life Technologies. GAPDH was used as an internal control to calculate the relative mRNA expression levels.

### 2.4. Western Blot Analysis

BMMSCs were washed and lysed, and the protein concentrations were measured using the BCA Protein Assay Kit (Thermo Scientific) with bovine serum albumin as the standard. Lysates (20 *μ*g/lane) were separated by 12% sodium dodecyl sulfate-polyacrylamide gel electrophoresis (SDS-PAGE); then, proteins were transferred to 0.22 *μ*m hybridization nitrocellulose filter membranes (Merck Millipore Ltd.). The membranes were blocked in TBST containing 5% nonfat milk (*w*/*v*) for 3 h at room temperature. Peripherin was detected by western blot using a peripherin primary antibody (rabbit anti-peripherin antibody (ab4666), Abcam). After washing with TBST, the membrane was incubated with the corresponding secondary antibody (goat anti-rabbit IgG H&L (HRP) (ab205718), Abcam). The immunoreactive bands were detected using SuperSignal West Pico Chemiluminescent Substrate (Thermo Scientific), recorded on X-ray films (CWBIO), and then analyzed using ImageJ software (NIH, Bethesda, MD, USA). The GAPDH antibody (Cell Signaling Technology #5174) was used to monitor variation in loading of samples.

### 2.5. Scratch Assay

The in vitro scratch assay is an easy, low-cost, and well-established method to measure cell migration in vitro. The process was the same as described previously [20]. The BMMSCs were seeded on 60 mm dishes and grown to confluence. The growth-arrested BMMSCs were transferred to a 6-well plate at a density of 3 × 10^6^ cells per well and incubated with 10 *μ*g/mL mitomycin-C for 2 h; then, the culture media were changed. After the BMMSCs were cultured for 6 h, the bottom of the plate was scratched using a sterile 10 ml pipette tip, producing a 1 mm wide area lacking cells; then, the BMMSCs were cultured for another 48 h. Images of the BMMSCs in the culture dishes were acquired using a phase contrast microscope at regular intervals (0 h, 6 h, 12 h, 24 h, 36 h, and 48 h).

### 2.6. Transwell Migration Assay

BMMSCs in 200 *μ*L of serum-free medium were reseeded on top of 5 *μ*m polycarbonate filter inserts in transwell chamber plates (Costar) at a density of 4 × 10^4^ cells/mL, and 800 *μ*L of culture medium containing 10% FBS was added into the lower compartments as a chemoattractant. Migration was allowed to proceed for 12 h at 37°C in a 5% CO_2_ incubator. After incubation, the filters were fixed with 4% paraformaldehyde, and the cells that had not migrated were removed from the upper surface of the filter by scraping using premium quality soft cotton buds. Then, the filters were stained with Hoechst 33342, washed with PBS, and subsequently observed under a fluorescence microscope (Nikon). The filters were visualized at 40x magnification, and the number of cells that had migrated across the filters was counted in at least nine random fields at the bottom of each filter.

### 2.7. Detection of the Rho Family Protein Activities

BMMSCs were cultured for 4 h in serum-free medium. Then, the cells were lysed in lysis buffer, and the activities of three Rho family proteins, RhoA-, Rac-1-, and Cdc42-GTP, were measured using G-LISA kits (Cytoskeleton) following the manufacturer's instructions.

### 2.8. F-Actin Staining

BMMSCs were grown on coverslips in 6-well plates for 24 h, incubated in media without FBS for 6 h, and then fixed with 4% paraformaldehyde and treated with 1% BSA+0.1% Triton at 4°C overnight. The following day, the cells were treated with 1 U/mL rhodamine-phalloidin for 30 min at room temperature, stained with DAPI (working concentration: 50 *μ*g/mL), and washed with PBS. The coverslips were mounted on slides and observed using an Axioskop 3-channel confocal microscope (Zeiss710). Images were obtained by *z*-scanning and were analyzed using Zen2010 software. At least three separate fields from triplicate wells for each genotype were analyzed per experiment.

### 2.9. Statistical Analysis

The experiment was repeated at least three times, and all data are presented as the mean ± SD. Statistical analyses were performed using Student's *t*-test. *P* < 0.05 was considered to indicate a statistically significant difference.

## 3. Results

### 3.1. Assessment of Specific Short Hairpin RNAs Targeting the PRPH Gene

**Figure 1:**
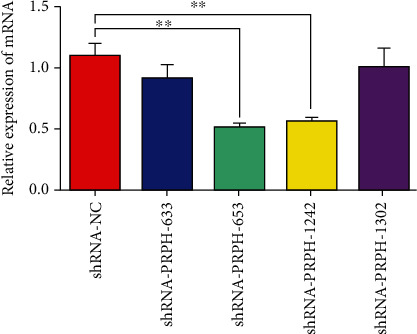
The expression levels of PRPH in the BMMSCs determined by qRT-PCR analysis. The relative expression levels of PRPH were measured in BMMSCs transfected with shRNA-NC, shRNA-PRPH-633, shRNA-PRPH-653, shRNA-PRPH-1242, or shRNA-PRPH-1302. The asterisk (∗∗) indicates a significant difference (*P* < 0.01) between groups.

RNA interference is the targeting of complementary RNAs for destruction using double-stranded RNAs. In mammalian systems, shRNAs can be expressed continuously to establish stable gene silencing. In this study, specific shRNAs directed against PRPH were used to knock down the PRPH expression in the BMMSCs from WZSP. After the BMMSCs were transfected with the shRNA-NC (control), shRNA-PRPH-633, shRNA-PRPH-653, shRNA-PRPH-1242, and shRNA-PRPH-1302 vectors, the levels of PRPH mRNA in the BMMSCs were evaluated by qRT-PCR assay. The results showed that the expression levels of PRPH in the BMMSCs transfected with shRNA-PRPH-653 and shRNA-PRPH-1242 were significantly lower than those in BMMSCs transfected with shRNA-NC (*P* < 0.01), indicating that the specific shRNAs directed against PRPH efficiently induced PRPH knockdown in the BMMSCs (Figure 1). The shRNA-PRPH-653 vector was the most efficient in inducing PRPH knockdown.

**Figure 2:**
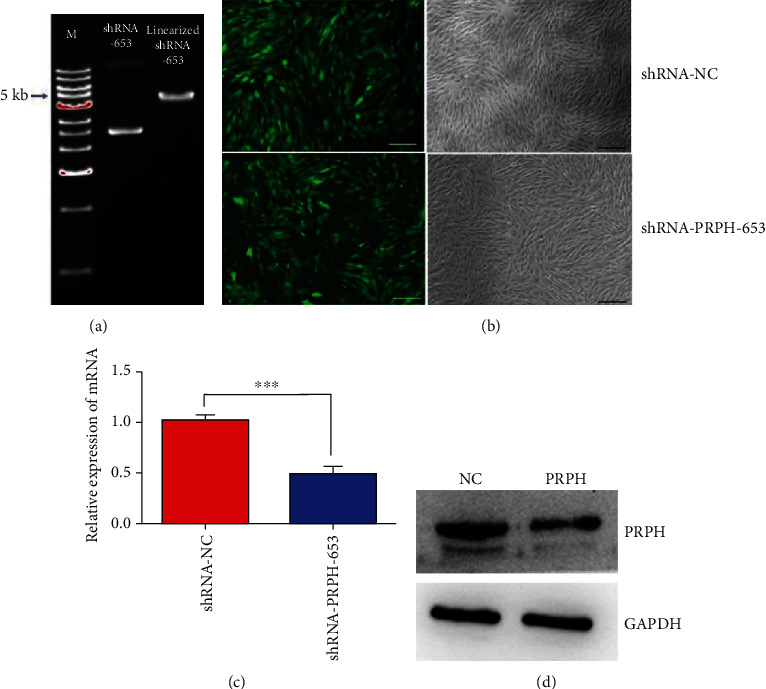
Effect of an shRNA specific for PRPH on the PRPH expression. (a) shRNA-PRPH-653 and linearized shRNA-PRPH-653 vectors were successfully constructed. (b) All BMMSCs were observed under a fluorescence microscope, and stable cell lines transfected with the shRNA-NC and shRNA-PRPH-653 vectors were obtained. Bar: 30 *μ*m. (c) Significant difference between the expression of PRPH in the shRNA-NC and shRNA-PRPH-653 groups determined by qRT-PCR analysis. (d) Analysis of the PRPH expression in the shRNA-NC and shRNA-PRPH-653 groups by western blot analysis. A GAPDH antibody was used to monitor variation in sample loading. Error bars represent the SEM (^∗∗∗^*P* < 0.001).

The shRNA-PRPH-653 and the linearized shRNA-PRPH-653 vectors were successfully constructed (Figure 2(a)). As illustrated in Figure 2(b), all BMMSCs were observed under a fluorescence microscope; stable cell lines transfected with the shRNA-NC and shRNA-PRPH-653 vectors were obtained after selection for 14 days, and nontransfected cells were removed. There was a significant difference in the expression of PRPH between the shRNA-NC and shRNA-PRPH-653 groups as determined by qRT-PCR analysis (*P* < 0.001) (Figure 2(c)), indicating that the PRPH expression was effectively knocked down in the BMMSCs transfected with shRNA-PRPH-653. Western blot analysis revealed an obvious decrease in the expression of peripherin in the shRNA-PRPH-653 group compared with the shRNA-NC group, but the GAPDH protein was expressed equally in both groups (Figure 2(d)).

### 3.2. The Role of Peripherin in BMMSC Migration

**Figure 3:**
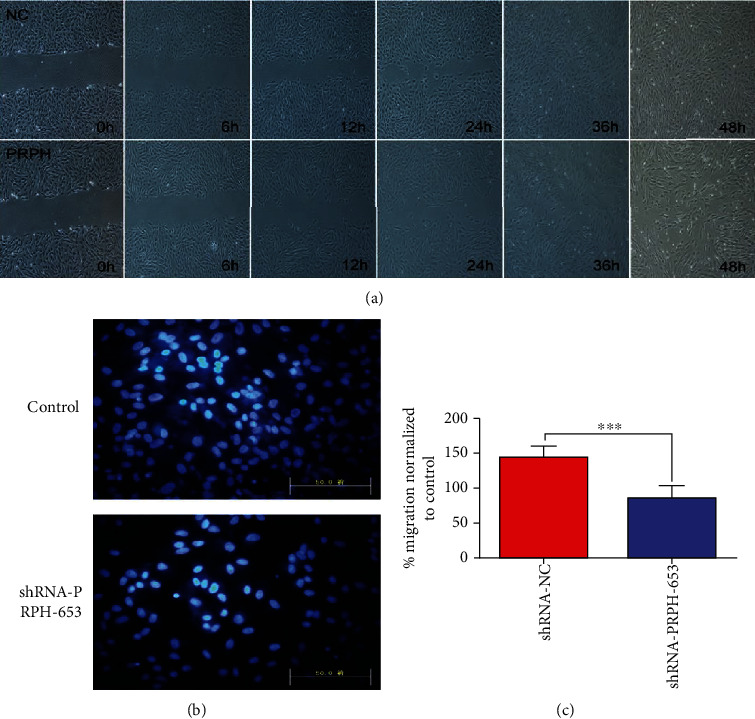
The ability of cells to migrate was measured by the scratch assay and transwell migration assay. (a) The migration capacity of BMMSCs analyzed by the scratch assay. A0, A6, A12, A24, A36, and A48 are images of the BMMSCs transfected with the shRNA-PRPH-653 vector acquired at 0 h, 6 h, 12 h, 24 h, 36 h, and 48 h after scratching, respectively. B0, B6, B12, B24, B36, and B48 are images of the BMMSCs transfected with the shRNA-NC (control) vector acquired 0 h, 6 h, 12 h, 24 h, 36 h, and 48 h after scratching, respectively. (b, c) The results of the BMMCS transwell assay. Control refers to BMMSCs transfected with the shRNA-NC vector. shRNA-PRPH-653 refers to BMMSCs transfected with the shRNA-PRPH-653 vector. Bar: 100 *μ*m. Error bars represent the SEM (^∗∗∗^*P* < 0.001).

To assess the migration of BMMSCs, a scratch was made across a layer of confluent BMMSCs using a pipette tip. Significant differences in migration ability between the shRNA-NC and shRNA-PRPH-653 BMMSCs were observed 12–24 h after scratching. Therefore, knocking down the PRPH expression may lead to a decreased ability of BMMSCs to migrate. The ability of BMMSCs to migrate was also investigated in transwell chambers with 5 *μ*m pores. After incubation for 6 h in the absence of any additional stimulus, the speed of BMMSC migration through the membrane was assessed under a biomicroscope (Figure 3(b)). The number of BMMSCs that migrated across the filters was 85.85 ± 2.55 with PRPH knockdown (shRNA-PRPH-653) but 143.47 ± 2.43 with the normal PRPH expression (shRNA-NC) (Figure 3(c)). Significantly, fewer BMMSCs migrated through the membrane in the shRNA-PRPH-653 group (*P* < 0.001, Figure 3(d)). The results of the scratch assay and transwell migration assay indicated that knockdown of the PRPH gene significantly decreased the migration capacity of BMMSCs.

### 3.3. PRPH Knockdown-Induced Change in Rho GTPase Activation

**Figure 4:**
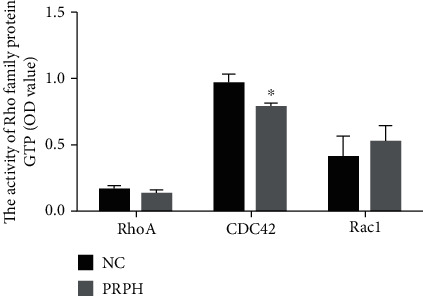
The GTPase protein activity of the Rho family (OD value). The shRNA-PRPH-653 BMMSCs displayed a large decrease in active Cdc42 (Cdc42-GTPase) levels compared with BMMSCs transfected with the control (NC). The activity of RhoA or Rac1 did not change. Error bars represent the SEM (^∗^*P* < 0.05).

As shown in Figure 4, after incubation in serum-free media for 6 h, shRNA-PRPH-653 BMMSCs displayed a large decrease in active Cdc42 (Cdc42-GTPase) levels. We also measured the levels of RhoA-GTP and Rac1-GTP in shRNA-PRPH-653 BMMSCs by ELISA. However, no significant change in the activity of RhoA or Rac1 was detected.

### 3.4. PRPH Knockdown-Induced Rearrangement of Filamentous Actin

**Figure 5:**
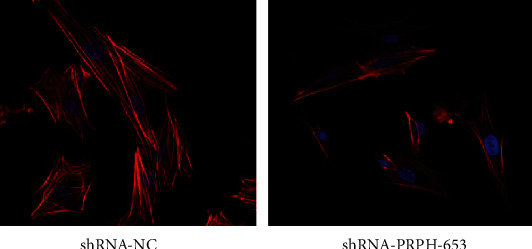
Imaging of F-actin in BMMSCs. The BMMSCs transfected with the shRNA-NC (control) vector formed longer microfilaments, while those transfected with the shRNA-PRPH-653 vector had shorter microfilaments and tended to become detached.

We examined the cytoskeletal organization in BMMSCs by rhodamine-phalloidin staining for 6 h in the absence of any additional stimulus. Confocal microscopy analysis of these cells showed that the control group BMMSCs displayed typical features of “nonpolarized” cells, and they had actin filaments that were significantly longer than those in shRNA-PRPH-653 BMMSCs and tended to become detached from the petri dish (Figure 5).

## 4. Discussion

BMMSCs are adult stem cells that have the capability to give rise to a variety of cells in the laboratory, including skeletal tissue, fat, and muscle cells [21]. However, the use of these cells for regenerative therapy needs more concern about safety, health, and ethical issues [22]. MSCs isolated from animals with less requirement of ethical aspects that were deemed as a good substitute of human MSCs had attracted more attention of researchers. BMMSCs from mini pigs have been investigated as models for in vitro research or as candidate donors for xenografting because they are easily handled, and less graft rejection occurs when their organs are used for transplantation [23–26].

In other proteomic studies, we identified peripherin as one of the proteins involved in the higher migration capability of BMMSCs. Peripherin encoded by PRPH is a type III intermediate filament protein found in neurons of the peripheral nervous system. Previous studies found that signals arising from peripherin-positive axons provide the appropriate guidance cues to LHRH cells as they migrate from the olfactory pit [27]. Therefore, we hypothesized that PRPH could affect the migration ability of BMMSCs from WZSP.

RNA interference (RNAi) is a natural cellular regulatory process that inhibits the gene expression through transcriptional, posttranscriptional, and translational mechanisms. Synthetic approaches that emulate this process use small interfering RNA (siRNA) and shRNA. shRNA transcribed from an expression vector intrinsically differs from synthetic double-stranded siRNA with respect to intracellular trafficking and nucleotide preference and can result in enhanced gene knockdown effects [19]. RNAi through the expression of shRNA is a promising approach for efficient gene silencing for therapeutic applications and a critical component of gene discovery [28]. In this study, in order to understand the role that PRPH plays in BMMSC migration, we used an shRNA approach to interfere with its expression [29]. The PRPH shRNA vector efficiently downregulated the PRPH expression in BMMSCs based on RT-PCR and western blot analysis, and we used the most efficient vector, shRNA-PRPH-653, to determine if PPRH influences the ability of BMMSCs to migrate.

Cell migration is essential in all multicellular organisms not only during development but also throughout the life cycle; migration is involved in processes such as wound repair and immune surveillance [30]. Although many studies have revealed factors involved in cell migration mechanisms, further study is essential to reveal more factors. In this study, we used the scratch assay and transwell migration assay to evaluate cell migration in vitro. The results of the scratch assay revealed that BMMSCs transfected with the shRNA-PRPH-653 vector reached confluence more slowly than those transfected with the control, shRNA-NC. The transwell migration assay demonstrated that fewer BMMSCs migrated when PRPH was knocked down. Furthermore, we showed that shRNA-PRPH-653 BMMSCs had significantly lower expression of Cdc42, a member of the Rho GTPase family that seems to play a pivotal role in regulating the biochemical pathways most relevant to cell migration [31, 32]. This finding provides indirect evidence of the effects of PRPH on cell migration.

It is now widely accepted that the major driving force underlying cell migration is the extension of a leading edge protrusion or lamellipodium, the establishment of new adhesion sites at the front of the cell, the rearrangement of F-actin, cell body contraction, and detachment of adhesions at the rear of the cell [31, 33]. In this study, we performed an F-actin assay and found that BMMSCs transfected with the shRNA-NC vector formed longer microfilaments, whereas the BMMSCs transfected with the shRNA-PRPH-653 vector displayed a more rounded morphology and had a tendency to become detached. These data support the role of PRPH in BMMSC migration.

## 5. Conclusions

Taken together, our results suggest that the PRPH gene could regulate BMMSC migration in WZSP. So far, to our knowledge, this is the first study reporting the influence of the PRPH gene on BMMSC migration in vitro. This study contributes to our understanding of the mechanism underlying MSC migration.

## Figures and Tables

**Table 1:** The sequences of specific short hairpin RNAs targeting PRPH.

Vector	Sequence name	Sequences
ShRNA-PRPH-633	S	5′-CACCGGAGCTAGAGCGCAAGATTCAAGAGGATCAATCTTGCGCTCTAGCTCCTTTTTTG-3′
	A	5′-GATCCAAAAAAGGAGCTAGAGCGCAAGATTGATCTCTTGAATCAATCTTGCGCTCTAGCTCC-3′
	Transcript	GGAGCTAGAGCGCAAGATTGATTCAAGAGATCAATCTTGCGCTCTAGCTCCTT
ShRNA-PRPH-653	S	5′-CACCGAGTCTTTGATGGATGAGATTTCAAGAGAATCTCATCCATCAAAGACTCTTTTTTG-3′
	A	5′-GATCCAAAAAAGAGTCTTTGATGGATGAGATTCTCTTGAAATCTCATCCATCAAAGACTC-3′
	Transcript	GAGTCTTTGATGGATGAGATTTCAAGAGAATCTCATCCATCAAAGACTCTT
ShRNA-PRPH-1242	S	5′-CACCGCATCCTTAAGTATAAAGACGATTCAAGAGATCGTCTTTATACTTAAGGATGTTTTTTG-3′
	A	5′-GATCCAAAAAACATCCTTAAGTATAAAGACGATCTCTTGAATCGTCTTTATACTTAAGGATGC-3′
	Transcript	GCATCCTTAAGTATAAAGACGATTCAAGAGATCGTCTTTATACTTAAGGATGTT
ShRNA-PRPH-1302	S	5′-CACCGCAAGATGGTTCTGATCAAGATTCAAGAGATCTTGATCAGAACCATCTTGCTTTTTTG-3′
	A	5′-GATCCAAAAAAGCAAGATGGTTCTGATCAAGATCTCTTGAATCTTGATCAGAACCATCTTGC-3′
	Transcript	GCAAGATGGTTCTGATCAAGATTCAAGAGATCTTGATCAGAACCATCTTGCTT

## Data Availability

The datasets used and/or analyzed during the current study are available from the corresponding author on reasonable request.
